# The *abcEDCBA*-Encoded ABC Transporter and the *virB* Operon-Encoded Type IV Secretion System of *Brucella ovis* Are Critical for Intracellular Trafficking and Survival in Ovine Monocyte-Derived Macrophages

**DOI:** 10.1371/journal.pone.0138131

**Published:** 2015-09-14

**Authors:** Auricelio A. Macedo, Ana P. C. Silva, Juliana P. S. Mol, Luciana F. Costa, Luize N. N. Garcia, Marcio S. Araújo, Olindo A. Martins Filho, Tatiane A. Paixão, Renato L. Santos

**Affiliations:** 1 Departamento de Clínica e Cirurgia Veterinárias, Escola de Veterinária, Universidade Federal de Minas Gerais, Belo Horizonte, MG, Brazil; 2 Departamento de Patologia Geral, Instituto de Ciências Biológicas, Universidade Federal de Minas Gerais, Belo Horizonte, MG, Brazil; 3 Centro de Pesquisas René Rachou, Fundação Oswaldo Cruz, Belo Horizonte, MG, Brazil; Universidad de Costa Rica, COSTA RICA

## Abstract

*Brucella ovis* infection is associated with epididymitis, orchitis and infertility in rams. Most of the information available on *B*. *ovis* and host cell interaction has been generated using murine macrophages or epithelial cell lines, but the interaction between *B*. *ovis* and primary ovine macrophages has not been studied. The aim of this study was to evaluate the role of the *B*. *ovis abcEDCBA*-encoded ABC transporter and the *virB* operon-encoded Type IV Secretion System (T4SS) during intracellular survival of *B*. *ovis* in ovine peripheral blood monocyte-derived macrophages. Δ*abcBA* and Δ*virB2* mutant strains were unable to survive in the intracellular environment when compared to the WT *B*. *ovis* at 48 hours post infection (hpi). In addition, these mutant strains cannot exclude the lysosomal marker LAMP1 from its vacuolar membrane, and their vacuoles do not acquire the endoplasmic reticulum marker calreticulin, which takes place in the WT *B*. *ovis* containing vacuole. Higher levels of nitric oxide production were observed in macrophages infected with WT *B*. *ovis* at 48 hpi when compared to macrophages infected with the Δ*abcBA* or Δ*virB2* mutant strains. Conversely, higher levels of reactive oxygen species were detected in macrophages infected with the Δ*abcBA* or Δv*irB2* mutant strains at 48 hpi when compared to macrophages infected with the WT strain. Our results demonstrate that *B*. *ovis* is able to persist and multiply in ovine macrophages, while Δ*abcBA* and Δ*virB2* mutations prevent intracellular multiplication, favor phagolysosome fusion, and impair maturation of the *B*. *ovis* vacuole towards an endoplasmic reticulum-derived compartment.

## Introduction

Brucellosis includes a group of diseases that results from infection of host species with different pathogens of the genus *Brucella* [1,2]. In most of the cases it is a zoonotic disease, which is associated with chronic infection in animals and humans. Brucellosis is responsible for great economic losses to the animal industry [3], and it is endemic in several countries, particularly in developing countries [4,5].

*Brucella ovis* is a stably rough Gram-negative coccobacillus, not encapsulated, not sporulated, that belongs to the alpha-2-Proteobacteria family [6]. Ovine brucellosis caused by *B*. *ovis* has worldwide distribution in all areas where sheep-raising has economic importance [7]. Infection with *B*. *ovis* is characterized by unilateral epididymitis in males (or bilateral, although less common), testicular degeneration and/or atrophy, that may result in subfertility, causing large economic losses [8–10]. In ewes, *B*. *ovis* infection can lead to abortion or birth of weak offspring, leading to perinatal death. Importantly, *B*. *ovis* is not been associated with human infections [11].

Several virulence factors of *Brucella* spp. that are important for the establishment of chronic infection in animals have been studied. For instance, the *virB* operon-encoded Type IV Secretion System (T4SS) is required for multiplication and intracellular survival, and it plays a critical role in intracellular trafficking of *Brucella* spp. The T4SS modulates maturation of the *Brucella*-containing vacuole (BCV), inhibiting its fusion with lysosomes, driving the pathogen towards a vacuole that contains features of the rough endoplasmatic reticulum, the intracellular multiplication niche of *Brucella* spp. [12–14]. Inactivation of the T4SS results in strong attenuation of *Brucella* spp., including *B*. *ovis*, either in infected cultures cells tissue explants *in vitro* or *in vivo* in animal models of infection [12,15–19].

Tsolis et al. [20] identified a genomic island on chromosome II of *B*. *ovis* that was absent in other classical *Brucella* species. The island was named *B*. *ovis* pathogenicity island 1 (BOPI-1), since it encodes genes that are associated with pathogenicity [21]. For instance, deletion of two genes encoding components of an ATP-binding cassette (ABC) transporter (BOV_A0504-BOV_A0500, named *abcEDCBA*) resulted in significant attenuation of *B*. ovis in mice [21] and rams [10]. It has recently been described that the encoded *abcEDCBA*-encoded ABC transporter interferes with the expression of the T4SS at a post-transcriptional level [22].

*Brucella* spp. has the ability to survive and multiply intracellularly in macrophages and non phagocytic cells [23]. Macrophages are key players for the innate immunity, but they also play a role in the development of acquired immune protective response against the *Brucella* [24]. However, macrophages are also important for the persistence of *Brucella* spp. in the host [25,26]. Therefore, experimental studies in primary macrophages infected with *Brucella* are important for a full understanding of the pathogenesis and virulence mechanisms of this bacterium, as well as the immune response of the host. Importantly, all published information regarding *B*. *ovis* pathogenesis is based on murine macrophages or epithelial cell lines [18,21,22], and there are no studies evaluating the interaction of *B*. *ovis* with macrophages from its preferential host, which is an essential step for validating experimental results obtained with cell lines particularly because *B*. *ovis* is one of the most host restricted *Brucella* spp.

The aim of this study was to evaluate the role of the T4SS and a species-specific ABC transporter in intracellular survival and trafficking of *B*. *ovis* in monocyte-derived macrophages from rams.

## Materials and Methods

### Bacterial strains, media and culture condition

The wild type *B*. *ovis* ATCC 25840 strain (WT), Δ*abcBA* mutant strain lacking a putative ABC transporter [21], and Δ*virB2* mutant strain lacking a functional T4SS [18] were used in this study (Table 1). WT *B*. *ovis*, Δ*abcBA* and Δ*virB2* isogenic strains expressing a *mCherry* fluorescent protein (named *mCherry-*WT *B*. *ovis*, *mCherry-*Δ*abcBA* and *mCherry-*Δ*virB2*, respectively) were used in this study (Table 1). These strains were constructed by the insertion of pKSoriT-*bla-kan-*P*sojA-mCherry* plasmid [27] adjacent to the constitutive promoter *secE* on chromosome I of *Brucella*. Inocula were cultured in Tryptone Soy Agar (TSA) (Invitrogen, USA) with 1% hemoglobin (Becton-Dickinson, USA) at 37°C in 5% CO_2_ for 3 days. Kanamycin (Kan, 100 mg/mL) and Ampicillin (Amp, 200 mg/mL) were added to media when necessary. For *mCherry-*WT *B*. *ovis*, *mCherry-*Δ*abcBA*, and *mCherry-*Δ*virB2* strains, selected colonies were Amp resistant and fluorescent, as previously described [27]. Bacterial suspensions were adjusted to final concentration by spectrophotometer at an optical density of 600 nm (OD600), serially diluted (10-fold) in phosphate-buffered saline (PBS), plated on TSA plates with 1% hemoglobin incubated at 37°C in 5% CO_2_ for 3 days and then the number of colony forming units (CFU) was calculated to confirm inoculum.

**Table 1:** Bacterial strains and plasmid used in this study.

Strains/Plasmid	Description	Reference
Strains		
WT *B*. *ovis*	*B*. *ovis* ATCC25840	ATCC
*B*. *ovis* Δ*abcBA*	*B*. *ovis* ΔBOV2_A500-501::Kan^R^	[21]
*B*. *ovis* Δ*virB2*	*B*. *ovis* Δ*virB2*:pAV2.2:: Kan^R^	[18]
WT *B*. *ovis*-*mCherry*	*B*. *ovis*:pKSoriT+mCherry-Kan^R^, Amp^R^	This study
*B*. *ovis* Δ*abcBA*-*mCherry*	*B*. *ovis* Δ*abcBA*:pKSoriT+mCherry- Kan^R^, Amp^R^	This study
*B*. *ovis* Δ*virB2-mCherry*	*B*. *ovis* Δ*virB2*: pKSoriT+mCherry- Kan^R^, Amp^R^	This study
Plasmid		
pKSoriT+mCherry	pKSoriT-bla-kan-carb-PsojA-mCherry	[27]

R: resistance

### Ovine monocyte-derived macrophages isolation, culture, and infection

Primary macrophage cultures were obtained by differentiation of monocytes derived from peripheral blood. Briefly, 60 mL of blood from adult rams belonging to the Veterinary School of the Universidade Federal de Minas Gerais, with a syringe containing acid-citrate-dextrose (ACD) by puncture of the jugular vein. Rams were considered free of *B*. *ovis* based on agar gel immunediffusion (AGID) and polymerase chain reaction (PCR). Initially, blood was diluted with an equal volume of PBS (pH 7.4), slowly deposited on a column of Histopaque solution (Sigma-Aldrich, USA), and then centrifuged at 1000 x g for 40 min at 18°C. After separation, the interface layer containing mononuclear cells was collected, washed three times in PBS, and re-suspended in 15 mL of RPMI (Invitrogen, USA) supplemented with 4 mM L-glutamine (Invitrogen, USA), 1 mM non-essential amino acids (Invitrogen, USA), 1 mM sodium pyruvate (Invitrogen, USA), 2.9 mM sodium bicarbonate, 10% of Fetal Bovine Serum (FBS) (Invitrogen, USA), and penicillin/streptomycin (100 IU/mL). Cells in suspension were transferred to 50-mL Teflon Erlenmeyer flasks (Nalgene Company, USA), and incubated at 37°C in 5% CO_2_ for 24 h. Non-adherent cells were removed, 15 mL of supplemented RPMI containing 10% FBS without antibiotics was added, and cells were incubated at 37°C in 5% CO_2_ for 11 days, changing the medium every 3 days. This experimental protocol has been approved by the Ethics Committee on Animal Use of the Universidade Federal de Minas Gerais (CEUA-UFMG, Protocol 62/2014).

After 11 days in culture, macrophages were removed from the flasks by cooling on ice for 20–30 min, followed by vigorous agitation. Cell suspension was transferred to sterile polypropylene tubes and centrifuged at 1000 x g for 10 min at 4°C. Cells were re-suspended in supplemented RPMI containing 10% FBS, and the number of viable cells was determined based on exclusion of trypan blue in a hemocytometer chamber. A cell suspension containing 5 x 10^4^ macrophages were seeded into 96-well plates (100 μL per well in supplemented RPMI containing 10% FBS), incubated overnight at 37°C in 5% CO_2_ and then inoculated with WT *B*. *ovis*, Δ*abcBA* or Δ*virB2* at a concentration of 5 x 10^7^ bacteria/mL with multiplicity of infection (MOI) of 100:1. The plate was centrifuged at 400 x g for 5 min at room temperature and incubated for 30 min at 37°C in 5% CO_2_. Then, the medium from each well was replaced with RPMI supplemented with 10% FBS and gentamycin (Invitrogen, USA) at a final concentration of 100 μg/mL and incubated for 1 h at 37°C in 5% CO_2_ for inactivation of extracellular bacteria. Additionally, bacterial suspension was incubated separately with RPMI medium containing gentamicin and the suspension plated on TSA plate with 1% hemoglobin, to confirm the activity of gentamicin. To assess internalization time (termed zero time point), the macrophages were washed once with PBS, lysed with sterile distilled water, serially diluted in PBS and plated onto TSA plates with 1% antibiotic-free hemoglobin (WT) or containing kanamycin 100 μg/mL (mutant) for CFU counts. To evaluate the kinetics of intracellular survival, macrophages were infected and lysed in the same conditions at 8, 24, and 48 h post infection (hpi). All these experiments were repeated three times including the *mCherry-*WT *B*. *ovis*, *mCherry-*Δ*abcBA* and *mCherry-*Δ*virB2*.

### Immunocytochemistry

Macrophages were seeded and infected with the WT *B*. *ovis*, Δ*abcBA* or Δ*virB2* in chamber slides (Nunc Lab-Tek II Chamber Slide System, Thermo Scientific, USA) with MOI 100:1, under the same conditions mentioned above. At 8, 24, and 48 hpi, samples were fixed with 4% paraformaldehyde in PBS for 20 min at room temperature and processed for immunocytochemistry. Briefly, each sample was washed 3 times with PBS and incubated for 30 min with primary antibody (1:5000) using serum from a rabbit experimentally inoculated twice (at a 1-month interval) with 1 x 10^9^ CFU of *B*. *ovis* (strain ATCC 25840) [21]. Then, slides were washed three times with PBS, incubated with secondary biotinylated antibody for 20 min, followed by washing again with PBS and incubated for 20 min with complex streptavidin-peroxidase commercial kit (LSAB kit, Dako Corporation, USA). The reaction was revealed with 3-amino-9-ethyl-carbazole (AEC) (Dako Corporation, USA) for 10 min and counterstained with Mayer's hematoxylin. The cells were evaluated for immunodetection of intracellular bacteria.

### Confocal microscopy

Macrophage was seeded overnight on 13-mm glass coverslips in a 24-well plate at a density of 6 x 10^5^ cells/mL and incubated overnight at 37°C in 5% CO_2_ to promote cell adhesion. For colocalization studies, macrophages were transiently transduced using a commercial kit CellLight Lysosome-GFP Beckman 2.0 (Invitrogen, USA) for a lysosomal associated membrane protein 1 (LAMP1) labeling or CellLight ER-GFP Beckman 2.0 (Invitrogen, USA) for a calreticulin (a endoplasmic reticulum marker) labeling, adjusted to the proportion of 30 viral particles per cell and incubated at 37°C in 5% CO_2_ for 16 h, according to the manufacturer's instructions. Macrophages were then infected with *mCherry-*WT *B*. *ovis*, *mCherry-*Δ*abcBA* and *mCherry-*Δ*virB2* with MOI 100:1, under the same conditions mentioned above. After 8, 24, and 48 hpi, each well containing a coverslip was washed three times with PBS, fixed with 4% paraformaldehyde in PBS for 20 min at room temperature under light protection. The coverslip was removed from each well and mounted on glass slide using mounting medium for fluorescence Fluoshield with 4'6-diamidino-2-phenylindole (DAPI) (Sigma-Aldrich, USA) and incubated for 5 min at room temperature under light protection. Samples were analyzed on a laser scanning confocal microscope LSM 510 Meta (Carl Zeiss, USA) equipped with an immersion objective of 63 x (Plan-Apochromat 63x/1.4). Confocal images of 1024 x 1024 pixels were acquired and gathered using Adobe Photoshop CS5. To quantify *B*. *ovis* infection in cells and LAMP1 or calreticulin positive compartment colocalization, at least 100 cells per sample were counted.

### Transmission electron microscopy (TEM)

Macrophages were seeded in chamber slides at concentration of 10^6^ macrophages/well and infected with WT *B*. *ovis*, Δ*abcBA* and Δ*virB2* in the concentration of 10^8^ bacteria/well (MOI 100:1), as previously described. Uninfected macrophages were included as controls in each time. After 30 min, 24 and 48 hpi, the macrophages were fixed with 2% glutaraldehyde in 0.1 M cacodylate buffer (pH 7.2) for 24 h and kept in 1 M cacodylate buffer (pH 7.2) until the processing for TEM. Samples were processed by the flat embedding technique described by Steiner et al. [28]. Ultrathin sections were made on an ultramicrotome (Leica EM UC6, Austria), counterstained with uranyl acetate and 5% lead citrate, and examined in a transmission electron microscope (Tecnai G2-12-12 kV—EIF SpiritBiotwin, USA).

### Measurement of nitric oxide and reactive oxygen species

Quantification of nitric oxide (NO) and reactive oxygen species (ROS) was based on fluorimetric quantification using the permeable fluorescent probe 4,5-diaminofluorescein diacetate (DAF-2 DA) (Sigma Aldrich, USA) and CellRox Deep Red Reagent (Invitrogen, USA), respectively. Briefly, macrophages were seeded in 96-well plates (7 x 10^4^ macrophages/well) and infected with WT *B*. *ovis*, Δ*abcBA*, or Δ*virB2*, and incubated for 8, 24, and 48 h, under the same conditions as described above. Lipopolysaccharide (LPS) from *Escherichia coli* (Sigma Aldrich, USA) at a concentration of 1 μg/mL in RPMI supplemented with 10% FBS was used to stimulate macrophages as positive control. Negative control consisted of non infected macrophages. The DAF-2 DA probes at a final concentration of 2.5 μM in PBS and CellRox at a final concentration of 5 μM in PBS were added separately to each well at each time point and incubated at 37°C in 5% CO_2_ for 1 h. After this time, the medium from each well was removed, and cells were fixed with 200 μL of 4% paraformaldehyde in PBS for 20 min at room temperature and protected from light. Then, the cells were resuspended from the plate by cooling on ice for 20–30 min, transferred to flow cytometry tubes and read on cytometer FACScalibur (Becton Dickinson, USA) using wavelengths of 488 nm/515 nm (excitation and emission, respectively) for DAF-2 DA and 644 nm/665 nm (excitation and emission, respectively) for CellRox Deep Red.

### Statistical analysis

All CFU data were logarithmically transformed and submitted to analysis of variance (ANOVA). For confocal microscopy, all percentage data were submitted to angular transformation prior ANOVA. Means of groups were compared by the Student Newman Keuls test (SNK), using statistical analysis program Graphpad Prism 5.0 (GraphPad Software, USA), and considered significant when p<0.05. All experiments were conducted in triplicate and data represent geometric mean and standard error of three independent experiments.

## Results

### Internalization and intracellular survival kinetics of WT *Brucella ovis*, Δ*abcBA* and Δ*virB2* in ovine macrophages

**Fig 1:**
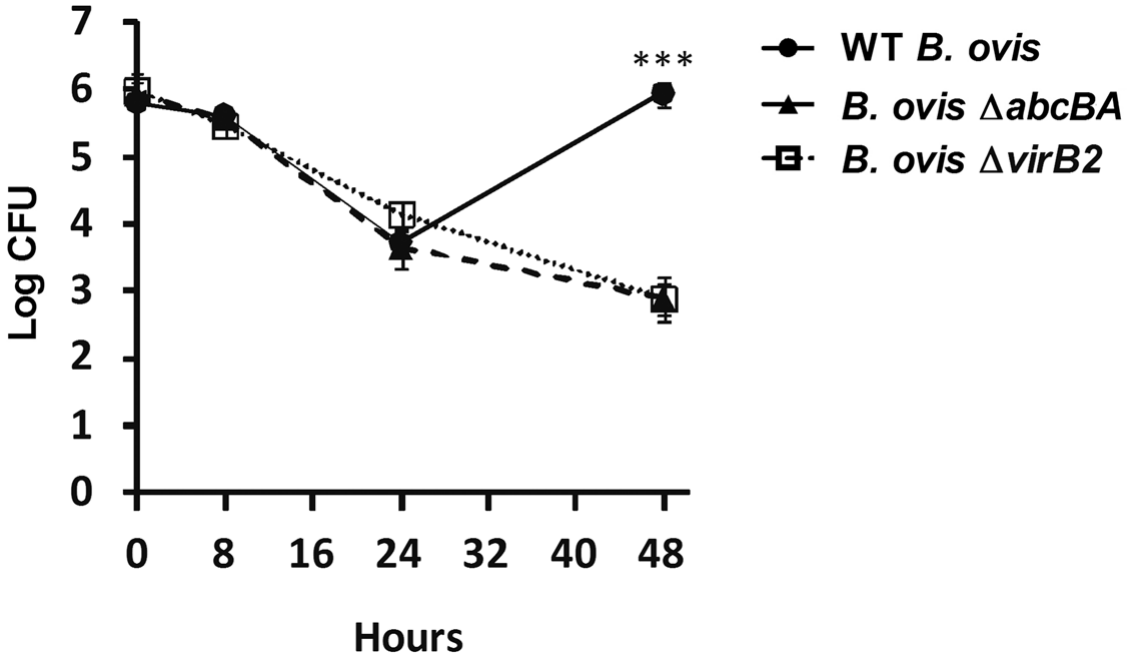
Internalization and intracellular survival of WT *B*. *ovis*, Δ*abcBA* and Δ*virB2* mutant strains in ovine macrophages. Data represent the geometric mean and the standard error (n = 6) from three independent experiments. Asterisk indicates statistically significant differences between the WT and mutant strains (*** p<0.001).

**Fig 2:**
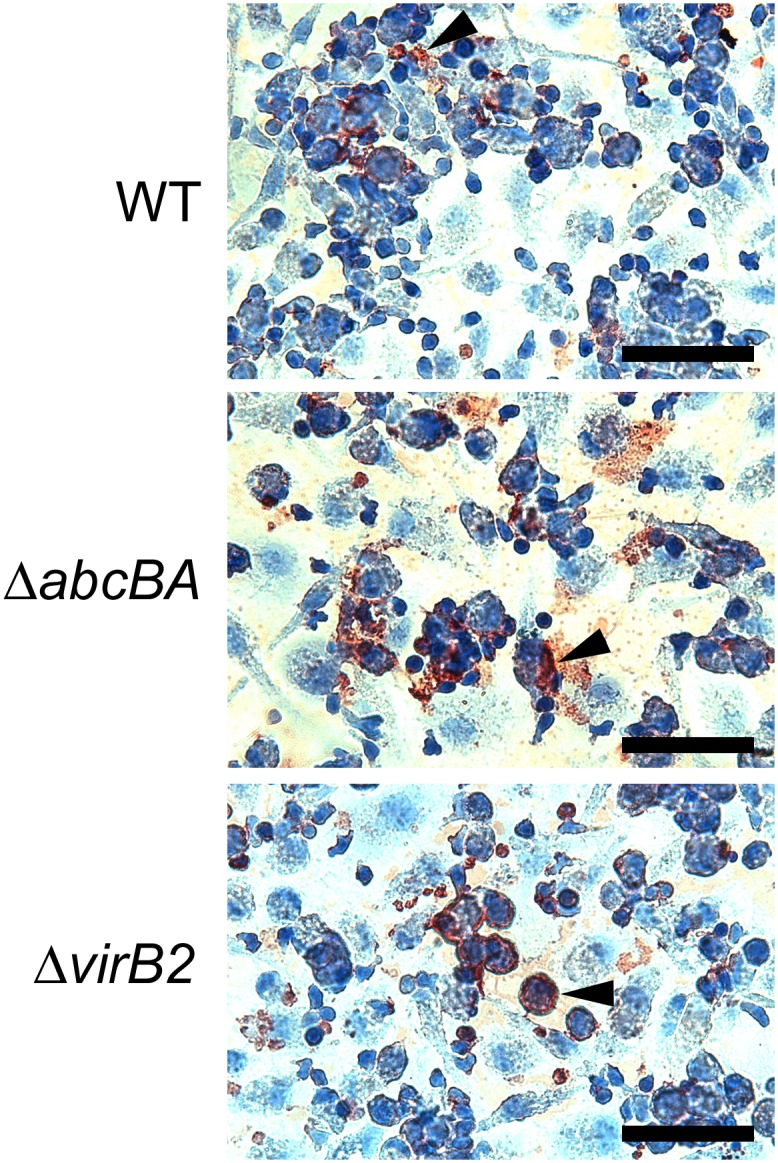
Representative image of intracytoplasmic immunostaining of WT *B*. *ovis*, Δ*abcBA* or Δ*virB2* mutant in ovine macrophages. Macrophages were infected in a chamber slide and then immunostained at 8, 24, and 48 h post infection. Immunostained WT, Δ*abcBA*, and Δ*virB2 B*. *ovis* (brown staining indicated by the arrowheads) localizes in the cytoplasm of ovine macrophages counterstained with Mayer’s hematoxylin. These are representative micrographs at 8 hpi. Bar = 50 μm.

Ovine peripheral blood monocyte-derived macrophages were infected with WT *B*. *ovis*, Δ*abcBA*, or Δ*virB2*, and intracellular bacteria were counted at 0, 8, 24, and 48 hpi (Fig 1). The internalization profile of WT *B*. *ovis*, Δ*abcBA*, and Δ*virB2* were similar (time 0), and CFU number decreased equally among the strains within 24 hpi. However, levels of infection of Δ*abcBA* and Δ*virB2* were significantly lower at 48 hpi (p<0.0001) when compared to WT *B*. *ovis*, confirming that these mutant strains are not able to multiply intracellularly in macrophages. In contrast, WT *B*. *ovis* was able to survive and multiply within ovine macrophages, with an increase in intracellular CFU numbers at 48 hpi (~2 logs) compared to the mutant strains. Immunocytochemical evaluation of macrophages after infection demonstrated localization of all bacteria strains in the cytoplasm of macrophages at all time points during the course of infection (Fig 2).

**Fig 3:**
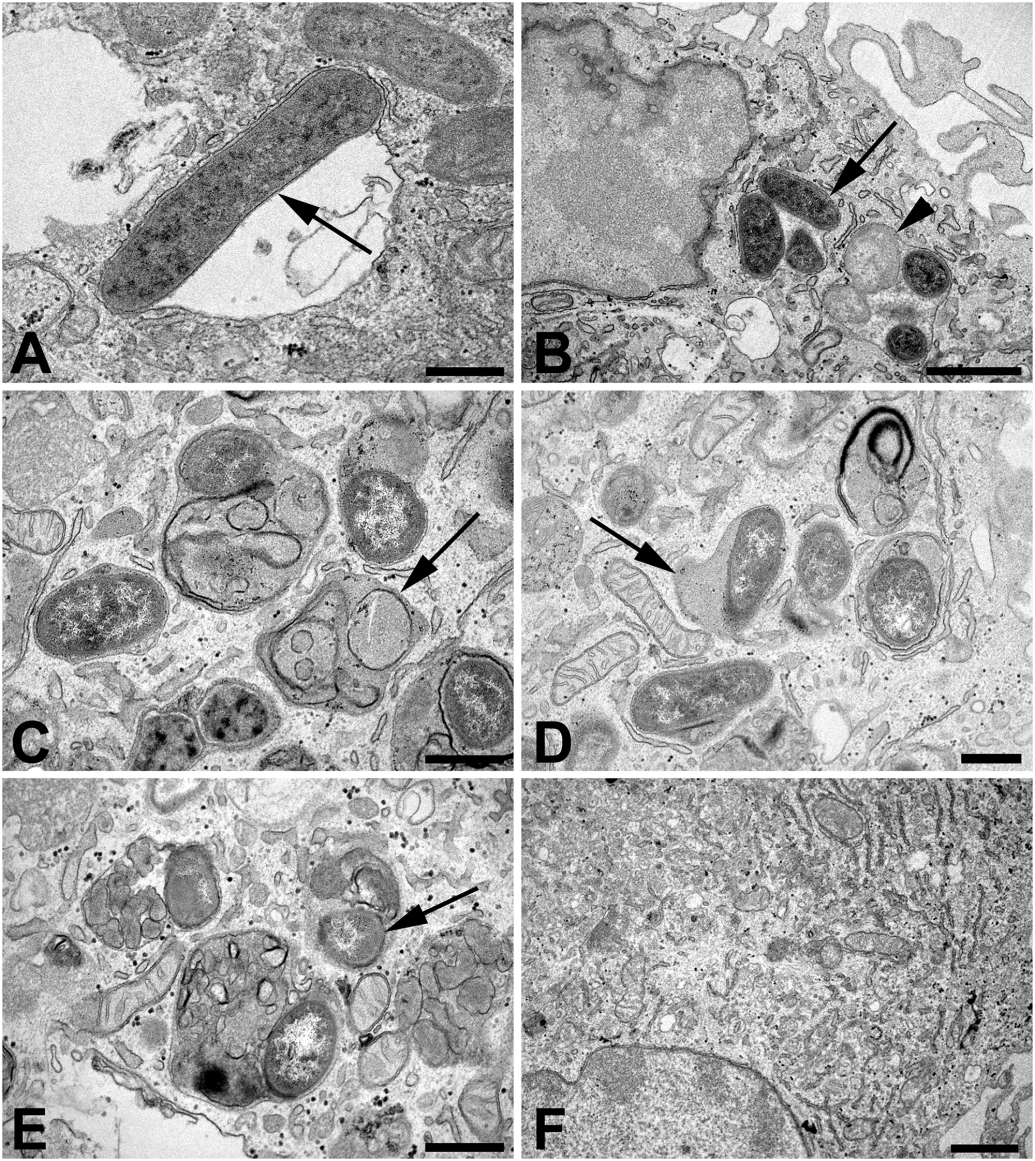
Transmission electron microscopy of ovine macrophages infected with WT *B*. *ovis*, Δ*abcBA* or Δ*virB2* mutant strains. Macrophages were infected for 30 min., 24 and 48 h in chamber slides, fixed with 2% glutaraldehyde in cacodylate buffer and processed for transmission electron microscopy. (A) Cytoplasmic vacuole containing a bacterium (arrow) in a macrophage infected with WT *B*. *ovis* at 30 min after infection; bar = 500 nm. (B) Vacuoles containing multiple bacteria (arrow) and degraded bacteria (arrow head) in a macrophage infected with *B*. ovis Δ*abcBA* at 30 min post infection; bar = 1 μm. (C) Myelin figures in a macrophage infected with *B*. *ovis* Δ*virB2* at 30 min post infection; bar = 500 nm. (D) Vacuole containing bacteria fused with lysosome, characterized by a mildly electrondense content within the vacuole (arrow), in a macrophage infected with *B*. *ovis* Δ*virB2* at 30 min post infection; bar = 500 nm. (E) Degenerated bacteria associated with myelin figures (arrow) in a macrophage infected with *B*. *ovis* Δ*virB2* at 24 h post infection; bar = 500 nm. (F) Macrophage infected with *B*. ovis Δ*abcBA* at 48 h post infection without any intracellular bacteria; bar = 1 μm.

Ultrastructural analysis of ovine macrophages following infection with WT *B*. *ovis*, Δ*abcBA*, or Δ*virB2* was performed at different time points after infection. At 30 min post infection, there were several bacteria within membrane bound vacuoles in macrophages infected with WT *B*. *ovis* (Fig 3A) or any of the mutant strains, although at 30 min post infection, there were evidences of bacterial degradation in vacuoles containing the mutant strains (Fig 3B). A few electrondense structures resembling folded membrane debris compatible with myelin figures were observed mostly in macrophages infected with the mutant strains (Fig 3C), which also had ultrastructural features of lysosome fusion as indicated by a slightly more electrondense contents within the bacteria containing vacuole (Fig 3D). At 24 hpi, there were larger numbers of degenerated bacteria within phagolysosomes or associated with myelin figures, mostly within macrophages infected with the mutant strains (Fig 3E), although degenerated bacteria were also observed in WT-infected macrophages. Importantly, myelin figures were not observed in non-infected macrophages (not shown). At 48 hpi, there were several bacteria inside membrane bound vacuoles in macrophages infected with WT *B*. *ovis*, whereas most of the macrophages infected with either one of the mutant strains (Δ*abcBA* or Δ*virB2*) no longer had intracellular bacteria (Fig 3F). No morphologic features of cell death were observed at any time point of infection with any of the *B*. *ovis* strains.

### Intracellular traffic of *mCherry-*WT *Brucella ovis*, *mCherry-*Δ*abcBA* and *mCherry-*Δ*virB2* in ovine macrophages

**Fig 4:**
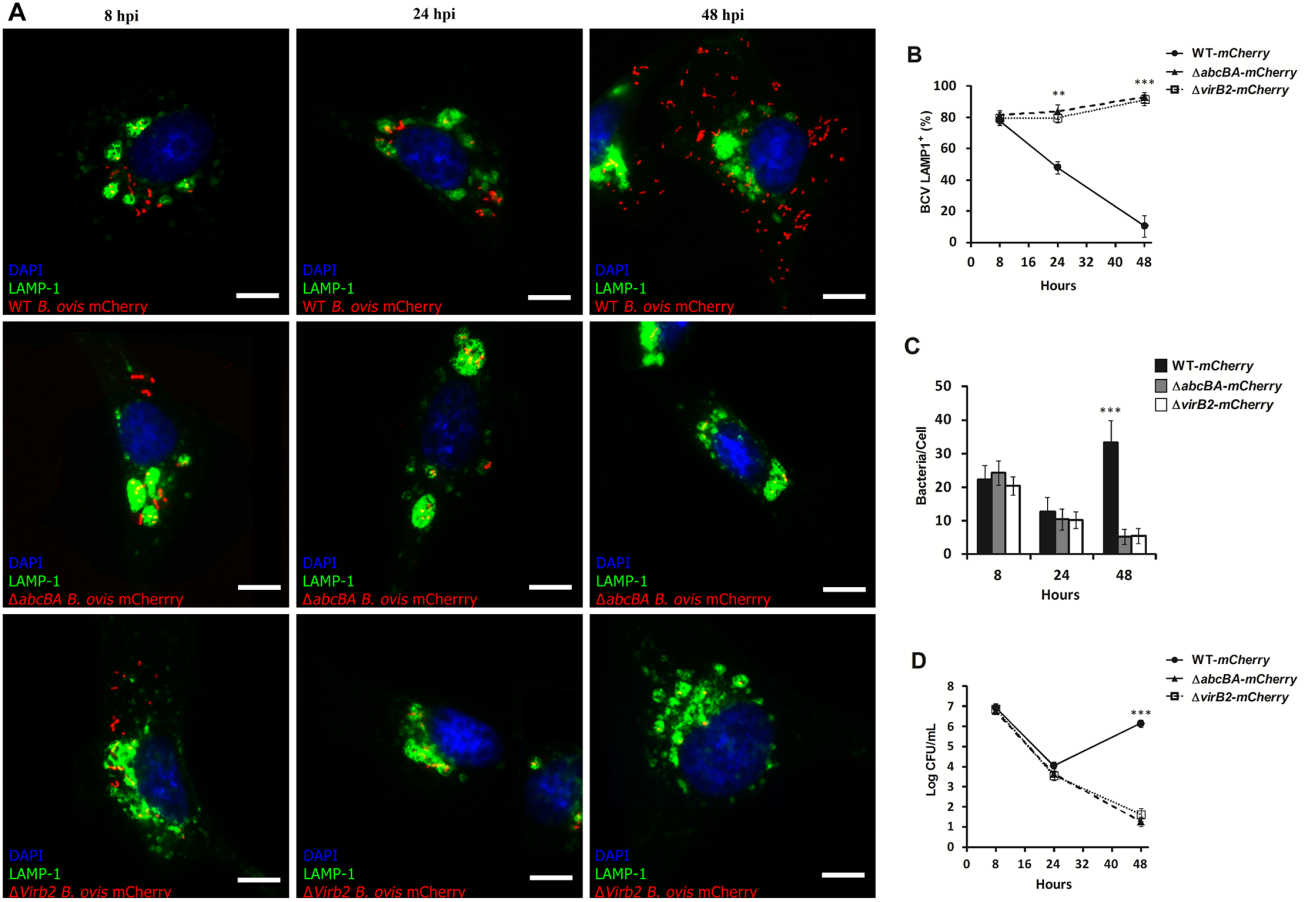
Intracellular trafficking of WT *B*. *ovis*, Δ*abcBA* and Δ*virB2* mutant strains in ovine macrophages. (A) Representative images of confocal microscopy of ovine macrophages expressing LAMP1 and infected with WT-*mCherry*, Δ*abcBA*-*mCherry* and Δ*virB2-mCherry* strains. (B) Average percentage of BCVs that were positive for LAMP1 during infection. (C) Average number of bacteria per cell (total of 100 cells per time point) during the course of the infection. (D) Kinetic infection of WT*-mCherry*, *ΔabcBA B*. *ovis*-*mCherry*, and Δ*virB2 B*. *ovis-mCherry* in ovine macrophages expressing LAMP1 by transient transduction (see Material and Methods section). Data represent mean and standard error of at least 100 cells from three independent experiments (** p<0.01; *** p<0.001).

Studies have shown the importance of the *abcEDCBA*-encoded ABC transporter and the T4SS for intracellular survival of *B*. *ovis* [18,21,22]. However, the role of these virulence factors in intracellular survival of *B*. *ovis* in macrophages from the preferential host of *B*. *ovis* has not been studied. To ensure that insertion of *mCherry* did not interfere with the *in vitro* growth and intracellular survival of *B*. *ovis* strains, an *in vitro* growth curve, and infection of macrophages were performed. As expected, the *mCherry-*WT *B*. *ovis*, *mCherry-*Δ*abcBA* and *mCherry-*Δ*virB2* strains grew similarly in TSA medium with 1% hemoglobin when compared to their *B*. *ovis* parental strains. As shown in S1 Fig, *mCherry-*WT *B*. *ovis*, *mCherry-*Δ*abcBA*, and *mCherry-*Δ*virB2* had kinetics of infection in ovine macrophages that were very similar to that of strains without insertion of *mCherry*. Due to the lack of commercially available cross-reacting antibodies to *Ovis aries* markers, we used a viral transduction-based method using a commercial kit CellLight Lysosome-GFP Beckman 2.0 (LAMP1 marker) and CellLight calreticulin-GFP Beckman 2.0 (endoplasmic reticulum marker), which allowed us to properly label these markers in ovine macrophages during the course of infection with *B*. *ovis* for evaluating intracellular trafficking of *B*. *ovis* in ovine macrophages. As shown in Fig 4A and 4B, LAMP1 was rapidly acquired by BCVs and most of the bacteria (~80%) colocalized with LAMP1^+^ compartments at 8 hpi. However, at 24 (p<0.001) and 48 hpi (p<0.0001), most BCVs from macrophages infected with *mCherry-*Δ*abcBA* and *mCherry-*Δ*virB2* were LAMP1^+^, in contrast to BCVs from macrophages infected with *mCherry-*WT *B*. *ovis*, which had a low percentage of colocalization. At 48 hpi, a large amount of *mCherry-*WT *B*. *ovis* was observed in macrophages when compared to the mutant strains *mCherry-*Δ*abcBA* and *mCherry-*Δ*virB2* (p<0.0001) (Fig 4C). To exclude the possibility that viral transduction could affect the kinetics of intracellular infection of the strains used in this study, internalization and intracellular survival experiments were repeated after transduction (Fig 4D). As expected, the number of CFU recovered over time was very similar to that shown in Fig 1.

**Fig 5:**
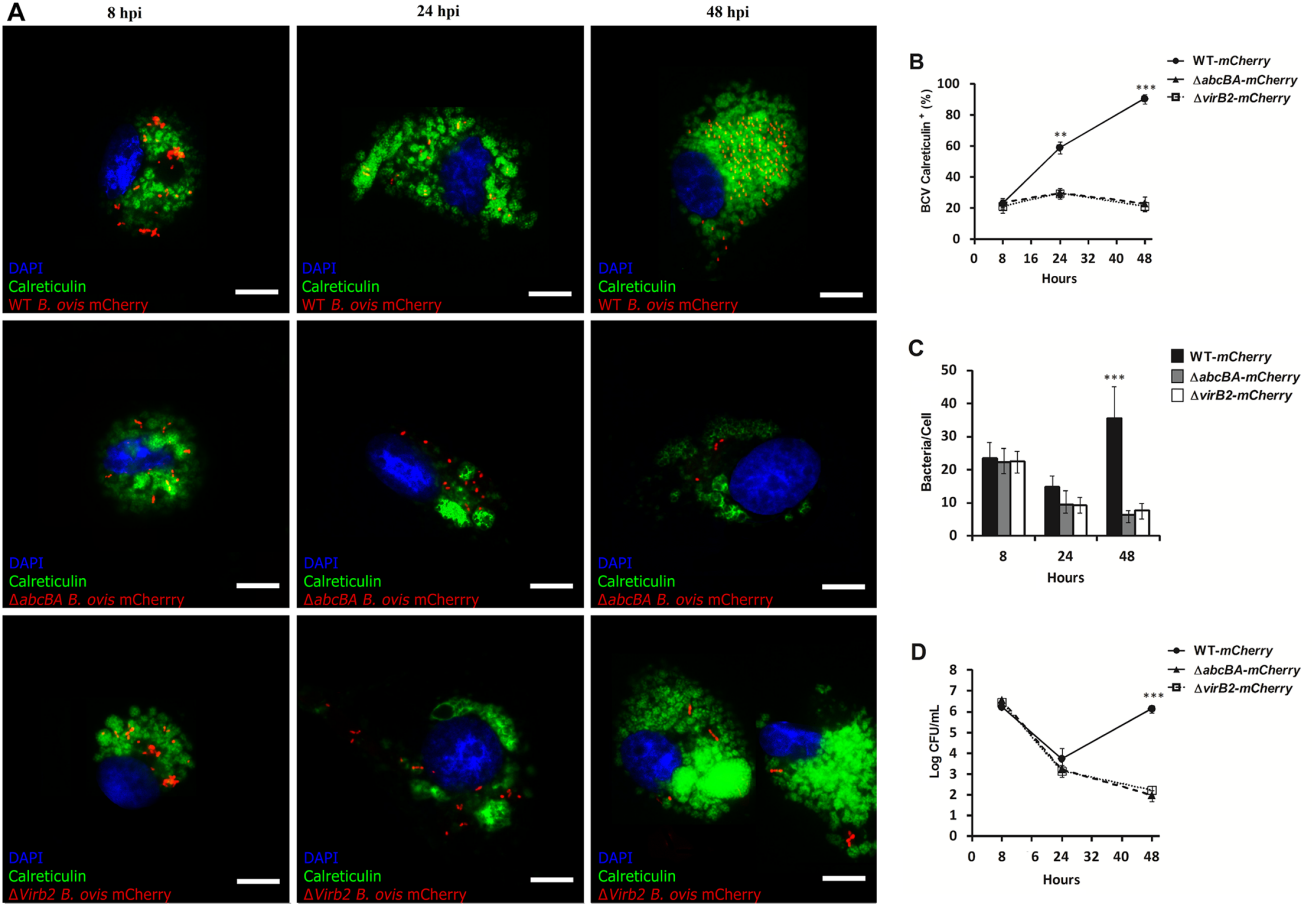
Intracellular trafficking of WT *B*. *ovis*, Δ*abcBA* and Δ*virB2* strains in ovine macrophages. (A) Representative images of confocal microscopy of ovine macrophages expressing Calreticulin and infected with WT-*mCherry*, Δ*abcBA*-*mCherry* and Δ*virB2-mCherry*. (B) Average percentage of BCVs that are positive for Calreticulin after infection. (C) Average number of bacteria counted per cell (total 100 cells per time) during the course of the infection. (D) Kinetic infection of WT*-mCherry*, *ΔabcBA B*. *ovis*-*mCherry* and Δ*virB2 B*. *ovis-mCherry* in ovine macrophages expressing Calreticulin by transient transduction (see Material and Methods section). Data represent mean and standard error of at least 100 cells from three independent experiments (** p<0.01; *** p<0.001).

In addition, similar confocal microscopy experiments with calreticulin labeling, an ER marker, were performed. At 8 hpi, a low percentage (~20%) of all bacteria (WT and mutants) colocalized with calreticulin^+^ compartments (Fig 5A and 5B). However, at 24 and 48 hpi, BCVs from macrophages infected with *mCherry-*WT *B*. *ovis* showed a progressive and extensive recruitment of calreticulin marker, which can be seen by the high percentage of colocalization, corresponding to approximately 60% and 90% of BCVs at 24 and 48 hpi, respectively. In contrast, BCVs from macrophages infected with the mutant strains *mCherry-*Δ*abcBA* or *mCherry-*Δ*virB2* had a very low percentage of colocalization with calreticulin over course of infection, which coincided with the low percentage of macrophages containing intracellular bacteria (~5%; p<0.0001) (Fig 5C). Similarly, viral transduction did not affect the kinetics of intracellular bacteria (Fig 5D). All together, these results demonstrate that *B*. *ovis* lacking ABC transporter or a functional T4SS are not able to control and direct the maturation of its vacuole into a multiplication niche, i.e. a vacuole similar to the ER.

### Nitric oxide and reactive oxygen species production in ovine macrophages infected with WT *Brucella ovis*, Δ*abcBA* or Δ*virB2* strains

**Fig 6:**
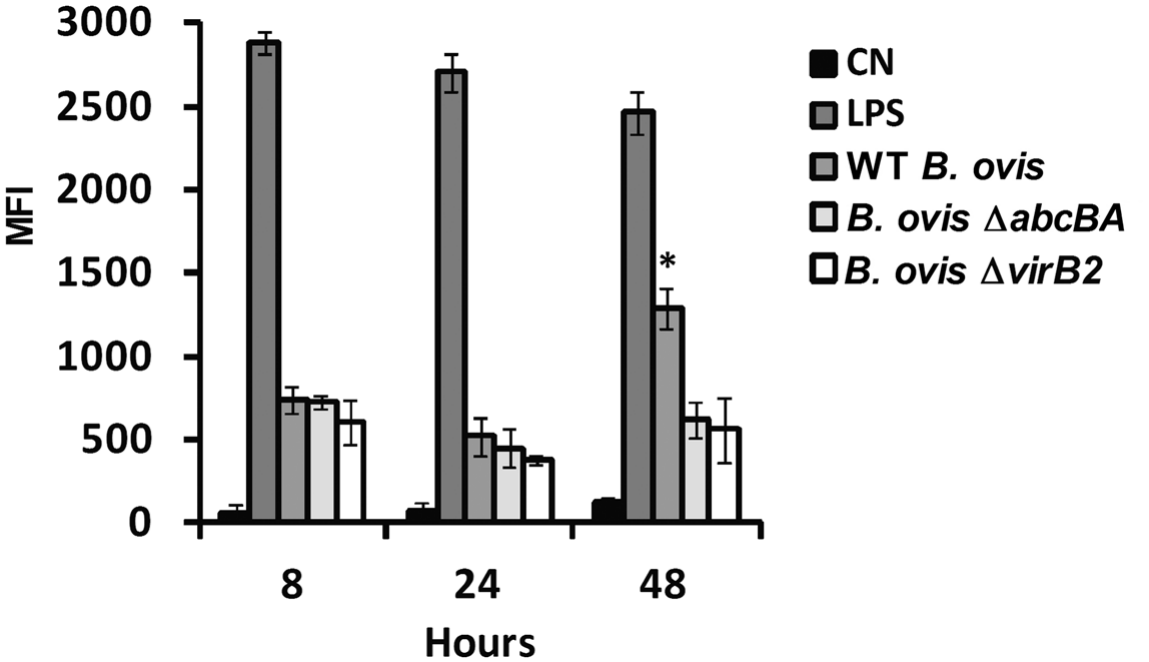
Production of NO by macrophages infected with WT *B*. *ovis*, Δ*abcBA* and Δ*virB2*. Macrophages were labeled with DAF-2 probe for 1 h and analyzed by flow cytometry. Results are expressed as mean fluorescent intensity (MFI). *E*. *coli* LPS (1 μg/mL) was used to stimulate macrophages as positive control. Not stimulated macrophages were used as negative control (NC). Bars represent mean and standard error of three independent experiments. Asterisk indicates statistically significant difference between WT and mutant strains (* p<0.05).

Nitric oxide (NO) production by ovine macrophages during the course of infection with WT *B*. *ovis*, Δ*abcBA* or Δ*virB2* was evaluated (Fig 6). As previously demonstrated, *E*. *coli* LPS induced high NO levels at 8, 24, and 48 hpi. NO was also detectable after infection with WT *B*. *ovis*, Δ*abcBA* or Δ*virB2* at all time points, although at lower levels when compared to LPS. Interestingly, at 48 hpi, WT *B*. *ovis* induced higher levels of NO when compared to Δ*abcBA* and Δ*virB2* mutant strains (p<0.01).

**Fig 7:**
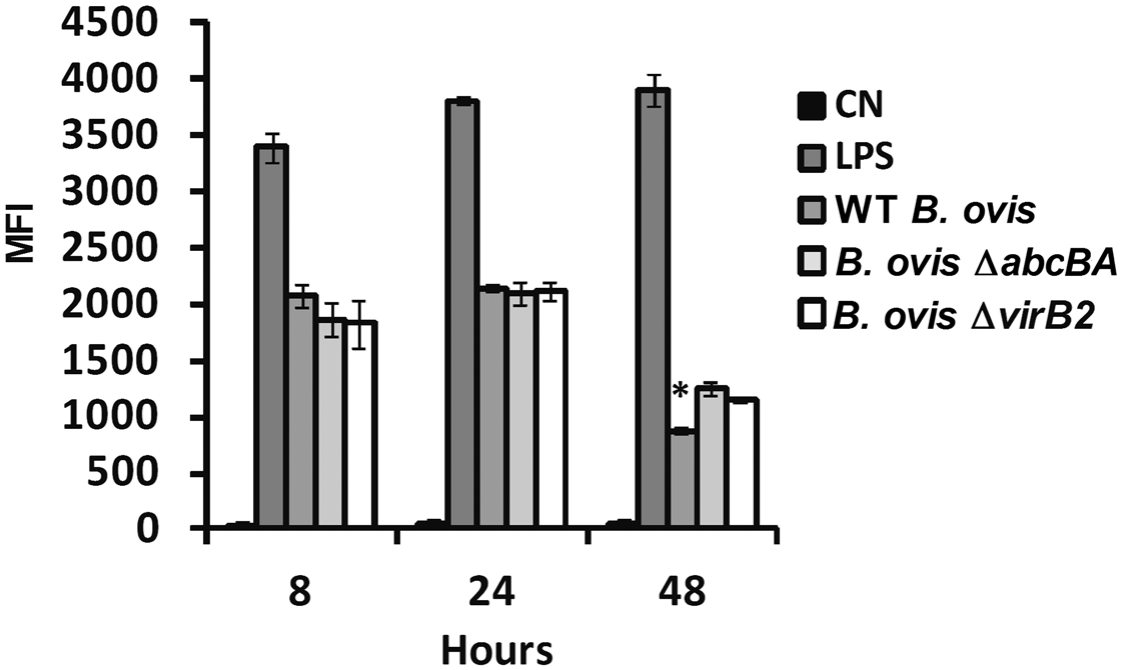
Production of ROS by macrophages infected with WT *B*. *ovis*, Δ*abcBA* or Δ*virB2* mutant strains . Macrophages were labeled with CellRox Deep Red probe for 1 h and analyzed by flow cytometry. Results are expressed as mean fluorescent intensity (MFI). *E*. *coli* LPS (1 μg/mL) was used to stimulate macrophages as positive control. Not stimulated macrophages were used as negative control (NC). Bars represent mean and standard error of three independent experiments. Asterisk indicates statistically significant difference between WT and mutant strains (* p <0.01).

Reactive oxygen species (ROS) production was also evaluated during the course of *B*. *ovis* infection in ovine macrophages. Likewise, *E*. *coli* LPS induced significant ROS production at all time points post infection. Higher levels of ROS were detected at 8 and 24 hpi in macrophages infected with WT *B*. *ovis*, Δ*abcBA* or Δ*virB2*, although no statistically significant differences between groups were observed. Interestingly, contrary to what was observed for NO, the Δ*abcBA* and Δ*virB2* mutant strains induced significantly higher amounts of ROS when compared to WT *B*. *ovis* (p<0.01) at time 48 hpi (Fig 7).

## Discussion

To the best of our knowledge, this is the first study describing intracellular survival and trafficking of *B*. *ovis* in primary ovine macrophages, i.e. phagocytes from the preferred host species of *B*. *ovis*. This is particularly important in the case of *B*. *ovis* since it is one of the most host restricted *Brucella* spp., which makes the evaluation of the interaction with primary macrophages from sheep crucial for validating previously published data on *B*. *ovis* pathogenesis. Our results demonstrated that Δ*abcBA* and Δ*virB2 B*. *ovis* strains are not able to interfere with intracellular trafficking and to multiply in ovine macrophages. These results are in good agreement with our previous studies, which demonstrated that Δ*abcBA* and Δ*virB2 B*. *ovis* strains are attenuated in cultured murine macrophages [18,21] or epithelial cells [22]. Our results also correlate well with the *in vivo* phenotype of the Δ*abcBA B*. *ovis* strain that is strongly attenuated in mice [21] and rams [10]. Interestingly, Silva et al. [22] demonstrated that even in non-phagocytic epithelial cell lines (HeLa cells) the Δ*abcBA B*. *ovis* strain was not able to multiply and survive in the intracellular environment. Importantly, there was no statistically significant difference in the internalization of Δ*abcBA* or Δ*virB2 B*. *ovis* when compared to the parental strain, suggesting that the putative ABC transporter as well as the T4SS play a role during the intracellular multiplication stage [14,18,21,22].

This is the first description of *B*. *ovis* trafficking in ovine monocyte-derived macrophages. This study confirmed that wild type *B*. *ovis* escapes from LAMP1^+^ compartment at early time points post infection, whereas the *B*. *ovis* vacuole acquires calreticulin marker, which is associated with intracellular multiplication at later time points. These findings are consistent to what has been shown for other species of the genus *Brucella*, either in phagocytic or non-phagocytic cells [13,14,22]. The *virB*-encoded T4SS of *Brucella* spp. is required for the vacuole maturation, which ultimately leads to acquisition of ER markers by the BCV, a crucial event for the biogenesis of the intracellular multiplication niche of *Brucella* [13].

During *Brucella* spp. intracellular trafficking, the early endosomal compartment interacts with lysosomes leading to acidification of the BCV, which induces T4SS expression by *Brucella*, a essential event for bacterial survival [13,29,30]. *B*. *ovis* expresses VirB proteins either in acidic or rich neutral media [22], which is in sharp contrast to other *Brucella* spp., suggesting an exclusive mechanism for T4SS regulation in *B*. *ovis*, independent of an acidic environment. Moreover, the deletion of the *B*. *ovis*-specific ABC transporter (Δ*abcBA*) interferes with expression of T4SS in a post-transcriptional level, in addition to other virulence factors such as expression of membrane protein Omp31 (BOV_1156), Cu-Zn Sod (SodC, BOV_A0659), and Fe-Mn Sod (SodB, BOV_0567) antioxidants [22]. However, despite a clear interference in protein biosynthesis of *B*. *ovis*, the *in vitro* growth of the mutant strain is not affected [21]. The BCV kinetic traffic in ovine macrophages are similar to that described for others cell lines, including epithelial cells, suggesting that *Brucella* uses similar mechanisms to control their intracellular survival and multiplication in different host cells [13,22,31].

In this study we found a clear association between *B*. *ovis* infection and the development of myelin figures, some of which associated with bacteria that were enclosed in multi-membrane vacuoles. These structures are morphologically compatible with autophagic compartments. Therefore, our findings provide support for the recently described pathway for intracellular survival of *Brucella abortus*, which subvert the host cell autophagic pathway [32], suggesting that *B*. *ovis* exploit the same mechanism for persistence in the intracellular environment, trafficking through a compartment that contains components of the rough endoplasmic reticulum, then moving towards a compartment with autophagic features [33] that may favor spreading from one host cell to the next [32].

The role of NO in the interaction between *Brucella* and macrophages during the course of infection remains controversial. Previous studies have reported a minor role of NO in controlling *B*. *abortus* infection in murine macrophages [34,35]. However, it has been reported that NO is an important anti-*Brucella* component, but only in pre-stimulated murine macrophages with INF-γ following opsonized-*Brucella* infection [34,35]. Our results indicated a low level of NO production throughout the course of infection and significant differences between WT *B*. *ovis* and Δ*abcBA* or Δ*virB2* only at 48 hpi, which coincides with the multiplication phase of WT *B*. *ovis*, suggesting that under *in vitro* conditions NO production seems to have no lethal effect on *Brucella*. For classical smooth *Brucella* species, such as *B*. *abortus* and *B*. *suis*, it is described that they can develop adaptive physiological changes that reduce their susceptibility to NO, which may explain why it can multiply even when NO is being produced [36,37]. Kikuchi et al. [38] demonstrated that the enzyme called D-alanyl-D-alanine carboxypeptidase (DAP) contributes to the NO resistance and is required for the intracellular growth of *B*. *abortus*. Conversely, higher levels of ROS were observed at the early stages of infection when the CFU numbers of WT or mutants are recovered in a smaller numbers, suggesting that *B*. *ovis* may be susceptible to bactericidal effect of ROS, as previously demonstrated for *B*. *abortus* [34,35]. NO and ROS are classified as effector molecules responsible for killing several intracellular pathogens including *Toxoplasma gondii* [39], *Leishmania* spp. [40,41], *Mycobacterium leprae* [42], *Mycobacterium tuberculosis* [43], and *Legionella pneumophila* [44]. However, microbes have developed mechanisms to subvert these host antimicrobial mechanisms. Some microbial molecules provide protection, including globins, flavorubredoxin, enzymes with NO- or S-nitrosothiol-reducing properties and other redox proteins. Flavohaemoglobin (Hmp) produced by *Escherichia coli*, *Mycobacterium bovis*, *Staphylococcus aureus*, *Salmonella* sp., and *Campylobacter jejuni*, is involved in NO detoxification and resistance [45,46]. Protozoal pathogens such as *Leishmania* sp. and *Trypanosoma cruzi* have developed mechanisms that prevent the microbicidal action of NO and ROS generated by host cells [47–49]. Similarly, mycotic infectious agents, including *Blastomyces dermatitidis*, *Coccidioides immitis*, *Paracoccidioides brasiliensis*, *Cryptococcus neoformans*, and *Histoplasma capsulatum*, also have the ability to prevent damage induced by NO in the intracellular environment, although the mechanisms involved in these cases are still unknown [50,51].

## Conclusion

In conclusion, our results demonstrated that *B*. *ovis* is able to persist and multiply in ovine macrophages and that deletion of the *B*. *ovis abcEDCBA*-encoded ABC transporter or inactivation of the T4SS prevent intracellular multiplication in ovine macrophages. These mutations (Δ*abcBA* and Δ*virB2*) prevent *B*. *ovis* evasion of phagolysosome fusion, and maturation of the vacuole towards an ER-derived compartment. Cultured ovine monocyte-derived macrophages proved to be a suitable model for studying *B*. *ovis* infection, host/pathogen interaction, and pathogenesis.

## Supporting Information

S1 Fig Kinetic infection in ovine macrophages of WT*-mCherry*, *ΔabcBA B*. *ovis*-*mCherry* and Δ*virB2 B*. *ovis-mCherry*. Data represent geometric mean and standard error (n = 6) from three independent experiments. Asterisk indicates statistically significant difference between the WT and mutant strains (*** p<0.001).
(TIF)
