# Supplementary figures and images for: The abcEDCBA-Encoded ABC Transporter and the virB Operon-Encoded Type IV Secretion System of Brucella ovis Are Critical for Intracellular Trafficking and Survival in Ovine Monocyte-Derived Macrophages
Source: PLoS One. 2015 Sep 14;10(9):e0138131. doi: 10.1371/journal.pone.0138131 (PMC4569489; doi:10.1371/journal.pone.0138131)

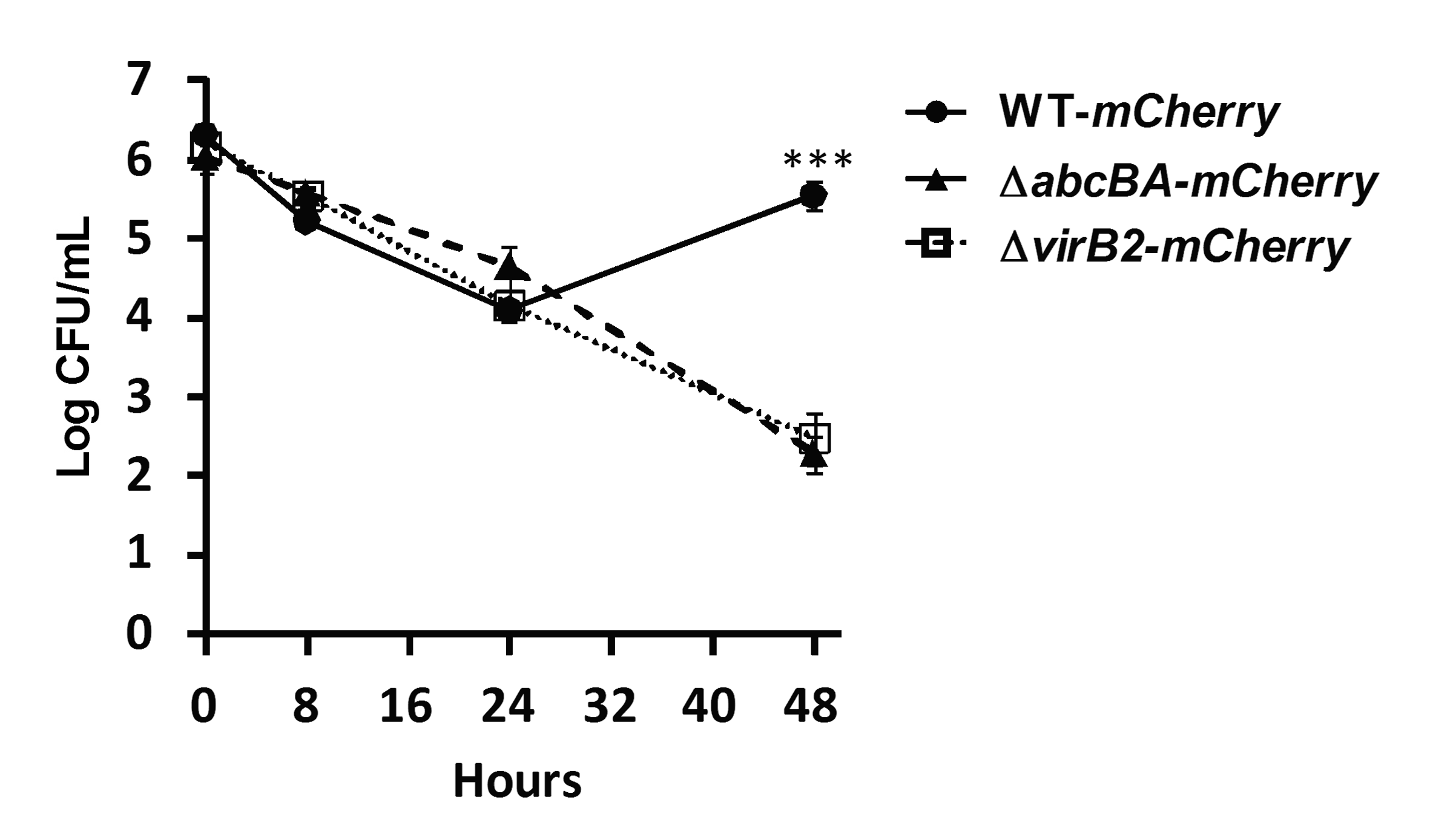

Supplement: S1 Fig — Data represent geometric mean and standard error (n = 6) from three independent experiments. Asterisk indicates statistically significant difference between the WT and mutant strains (*** p<0.001). (TIF) [file pone.0138131.s001.tif]
